# Enzyme replacement in the CSF to treat metachromatic leukodystrophy in mouse model using single intracerebroventricular injection of self-complementary AAV1 vector

**DOI:** 10.1038/srep13104

**Published:** 2015-08-18

**Authors:** Kohei Hironaka, Yoshiyuki Yamazaki, Yukihiko Hirai, Motoko Yamamoto, Noriko Miyake, Koichi Miyake, Takashi Okada, Akio Morita, Takashi Shimada

**Affiliations:** 1 Department of Biochemistry and Molecular Biology; Division of Gene Therapy, Research Center for Advanced Medical Technology; Nippon Medical School, Tokyo, 113-8603, Japan; 2 Department of Neurological Surgery; Nippon Medical School, Tokyo, 113-8603, Japan

## Abstract

Metachromatic leukodystrophy (MLD) is a lysosomal storage disease caused by a functional deficiency in human arylsulfatase A (hASA). We recently reported that ependymal cells and the choroid plexus are selectively transduced by intracerebroventricular (ICV) injection of adeno-associated virus serotype 1 (AAV1) vector and serve as a biological reservoir for the secretion of lysosomal enzymes into the cerebrospinal fluid (CSF). In the present study, we examined the feasibility of this AAV-mediated gene therapy to treat MLD model mice. Preliminary experiments showed that the hASA level in the CSF after ICV injection of self-complementary (sc) AAV1 was much higher than in mice injected with single-stranded AAV1 or scAAV9. However, when 18-week-old MLD mice were treated with ICV injection of scAAV1, the concentration of hASA in the CSF gradually decreased and was not detectable at 12 weeks after injection, probably due to the development of anti-hASA antibodies. As a result, the sulfatide levels in brain tissues of treated MLD mice were only slightly reduced compared with those of untreated MLD mice. These results suggest that this approach is potentially promising for treating MLD, but that controlling the immune response appears to be crucial for long-term expression of therapeutic proteins in the CSF.

Metachromatic leukodystrophy (MLD) is a rare autosomal recessive lysosomal storage disease (LSD) caused by deficient activity of a lysosomal enzyme, arylsulfatase A (ASA). ASA deficiency results in accumulation of the undigested substrate, sulfatide, in oligodendroglial and Schwann cells, leading to demyelination in the central and peripheral nervous systems^1^. Although some LSDs have been successfully treated using systemic enzyme replacement therapy (ERT)^2^ in which lysosomal enzymes injected into the patient’s circulation are taken up by target cells via a receptor-mediated pathway followed by cross-correction of the enzyme deficiency^3^, the clinical efficacy of ERT for LSD with neurological symptoms, including MLD, is very limited because lysosomal enzymes cannot cross the blood-brain barrier^4^. For delivery of therapeutic enzymes into the central nervous system (CNS) to treat neurological manifestations of LSD in patients, alternative drug delivery strategies to circumvent the blood-brain barrier are required.

One possible approach is direct injection of enzyme into the cerebrospinal fluid (CSF) that circulates throughout the CNS. Intra-CSF ERT corrects the CNS pathology and behavioral dysfunction in MLD mice^5^, and a phase I/II clinical trial of intrathecal ERT for MLD patients is currently ongoing (NCT01510028; http://clinicaltrials.gov). However, repeated infusion of enzyme into the CSF will impose a therapeutic and economic burden on patients over their entire lifespan. In such cases, gene therapy could help to reduce this burden by transducing cells within the CNS that will then continuously secrete therapeutic enzymes into the CSF for sustained periods.

Gene therapies with intracerebroventricular (ICV) injection of adeno-associated virus (AAV) encoding therapeutic enzymes correct the CNS pathology of LSD in model animals^6^,^7^,^8^,^9^. For example, intra-CSF administration of AAV serotype 9 (AAV9) encoding sulfamidase raises the levels of sulfamidase in the CSF and corrects both the CNS and somatic pathology of mucopolysaccharidosis type IIIA in model dogs^6^. On the other hand, we demonstrated recently that an ICV injection of AAV serotype 1 (AAV1) encoding human ASA (hASA) led to widespread expression of hASA in ventricular cells including ependymal cells and the choroid plexus^10^. Continuous secretion of hASA into the CSF of wild-type (C57BL/6J) mice was seen, and the levels were sustainable, similar to sustained secretion of sulfamidase in mucopolysaccharidosis type IIIA dogs^6^. Because the safety of AAV1 has been well characterized in several clinical trials^11^, testing of whether intra-CSF administration of AAV1 compared with other serotypes provides a sufficient source of therapeutic protein within the CNS of LSD patients to ameliorate their neurological pathology is important. Thus, in the present study, we compared the transduction efficacy in ventricular cells between AAV1 and AAV9 and assessed the effectiveness of this gene therapy in MLD mice using ICV injection of AAV1. Furthermore, prior to these studies, the transduction efficacy of ICV-injected vectors was compared between the two types of genome packaging, self-complementary AAV (scAAV) and single-stranded AAV (ssAAV). As second-strand DNA synthesis is a limiting factor for transgene expression after infection with AAV vectors^12^, we expected that scAAV may provide more effective transduction activity than ssAAV even when they are injected by the ICV route. Here, we show that the hASA level in the CSF after ICV injection of scAAV1 was much higher than levels following injection of ssAAV1 and scAAV9. In addition, we report that ICV injection of scAAV1 slightly reduced the sulfatide levels in the brain tissues of treated MLD mice compared with sulfatide levels in untreated MLD mice. Our results suggest that this AAV1-mediated ventricular cell transduction protocol is potentially promising for the treatment of LSDs with severe CNS involvement.

## Results

### CSF levels of hASA in mice that received an ICV injection of scAAV1 are higher than in those receiving ssAAV1

**Figure 1:**
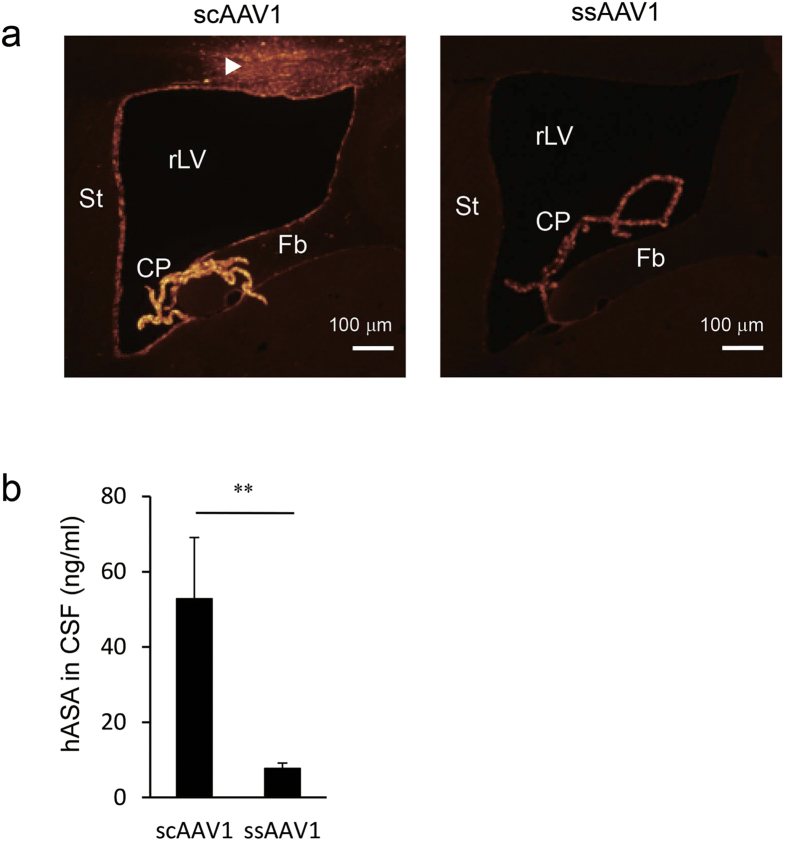
Expression of hASA induced by ICV injection of scAAV1-hASA was higher than that of ssAAV1-hASA in the brains of MLD mice at 2 weeks after injection. (**a**) Comparison of expression patterns of hASA between brains of mice given scAAV1-hASA (2.3 × 10^11^ vg) and ssAAV1-hASA (2.3 × 10^11^ vg) injection. Human ASA (red) expressed in mouse brain was stained immunohistochemically. The injection needle track is indicated by the arrowhead. CP, choroid plexus; Fb, fimbria; rLV, right lateral ventricle; St, striatum. (**b**) Comparison of hASA levels in CSF between mice given scAAV1-hASA (*n* = 6) and ssAAV1-hASA (*n* = 7) injection. Error bars indicate SEM values. The asterisks indicate a significant difference between the two groups (**P < 0.01, Mann-Whitney test).

First, we compared the transduction efficacy of scAAV and ssAAV vectors in ventricular cells following ICV injection. Eight-week-old MLD mice were given a unilateral ICV injection of 2.3 × 10^11^ vector genome (vg) of scAAV1 encoding hASA (scAAV1-hASA; *n* = 6) or ssAAV1-hASA (*n* = 7), both containing identical hASA expression cassettes. At 2 weeks after injection, mice were anesthetized, the cisterna magna was punctured to collect CSF samples, and the mice were sacrificed to obtain brain tissues for immunohistochemical and biochemical analysis (Fig. 1). Figure 1a shows immunohistochemical staining with an antibody against hASA in the brain sections of mice following injection of scAAV1-hASA or ssAAV1-hASA. Expression of hASA was observed in the choroid plexus and ependymal cells and seemed to be stronger in the brain injected with scAAV1-hASA compared to brains injected with ssAAV1-hASA at 2 weeks after injection. In brains injected with scAAV1-hASA, expression of hASA was also observed in the brain parenchyma near the injection site (arrowhead in Fig. 1a) and in Purkinje cells in the cerebellum (data not shown). Consistent with the results of immunohistochemical staining, an enzyme-linked immunosorbent assay (ELISA) showed that secreted hASA concentrations in the CSF of mice injected with scAAV1-hASA were significantly higher than those injected with ssAAV1-hASA (Fig. 1b; 52.8 ± 16.3 vs. 7.7 ± 1.4 ng/ml, respectively; *P* < 0.01, Mann-Whitney test). No background values were detected in the CSF of untreated MLD mice because the ELISA for hASA is highly specific. hASA was not detected in the blood plasma of any mice injected with ssAAV1-hASA or scAAV1-hASA (data not shown). Thus, we decided to use the scAAV vector for the next studies.

### CSF levels of hASA in mice that received an ICV injection of scAAV1 are higher than those receiving scAAV9

**Figure 2:**
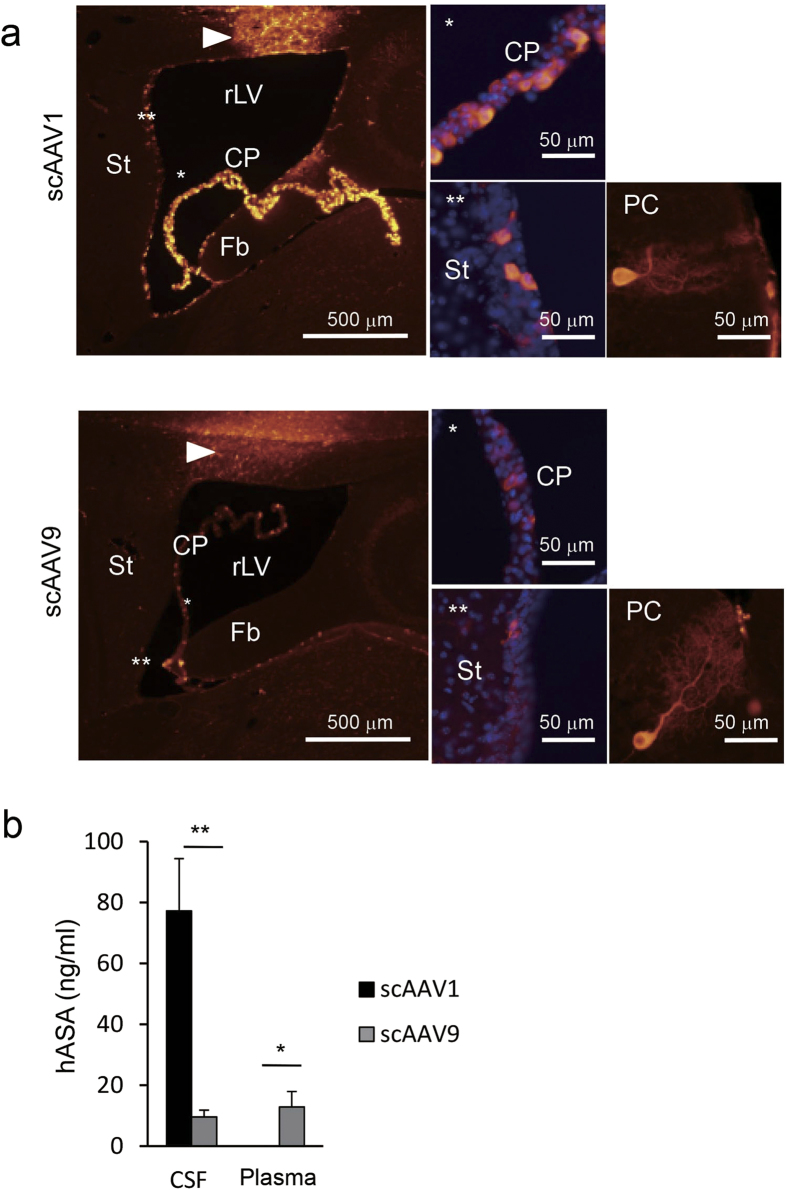
Expression of hASA induced by ICV injection of scAAV1-hASA was higher than that of scAAV9-hASA in the brains of wild-type mice at 2 weeks after injection. (**a**) Comparison of expression patterns of hASA between brains of mice given scAAV1-hASA (1.1 × 10^11^ vg) and scAAV9-hASA (1.1 × 10^11^ vg) injection. Human ASA (red) expressed in mouse brain was stained immunohistochemically. The injection needle tracks are indicated by arrowheads. Cell nuclei are stained with DAPI (blue) in higher magnification images of the choroid plexus (*) and ependymal cells (**). PC, Purkinje cell in the cerebellum. (**b**) Comparison of hASA levels in the CSF and plasma between mice given scAAV1-hASA (*n* = 6) and scAAV9-hASA (*n* = 6) injection. Error bars indicate SEM values. The asterisks indicate significant differences between the two groups (*P < 0.05, **P < 0.01, Mann-Whitney test).

Next, we compared the transduction efficacy of scAAV1 and scAAV9 in ventricular cells following ICV injection. Eight- to 9-week-old wild-type mice were given a unilateral ICV injection of 1.1 × 10^11^ vg of scAAV1-hASA (*n* = 6) or scAAV9-hASA (*n* = 6), both containing identical hASA expression cassettes. At 2 weeks after injection, blood plasma samples, CSF samples, and brain tissues were collected for immunohistochemical and biochemical analysis (Fig. 2). Figure 2a shows immunohistochemical staining with an antibody against hASA in the brain sections of mice following injection of scAAV1-hASA or scAAV9-hASA. Expression of hASA was observed in the choroid plexus, ependymal cells, Purkinje cells, and brain parenchyma near the injection site and seemed to be stronger in brains injected with scAAV1-hASA than in those injected with scAAV9-hASA at 2 weeks after injection. Consistent with the results of immunohistochemical staining, ELISA showed that hASA concentrations in the CSF of mice injected with scAAV1-hASA were significantly higher than those injected with scAAV9-hASA (Fig. 2b; 77.3 ± 17.1 vs. 9.6 ± 2.3 ng/ml, respectively; *P* < 0.01, Mann-Whitney test). The amount of hASA in the plasma may not be positively correlated with the concentration of hASA in the CSF, because the plasma levels of hASA were higher in the mice injected with scAAV9-hASA than in those injected with scAAV1-hASA (Fig. 2b; 12.8 ± 5.1 vs. 0 ± 0 ng/ml, respectively; *P* < 0.05, Mann-Whitney test).

**Figure 3:**
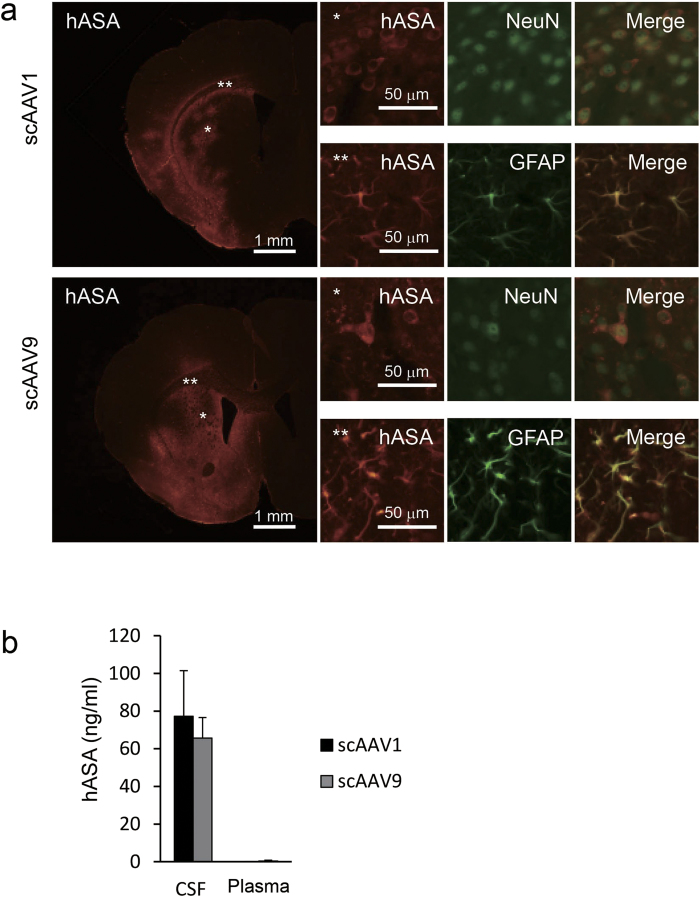
Expression of hASA induced by intrastriatal injection of scAAV1-hASA was nearly equal to that following injection of scAAV9-hASA in the brains of wild-type mice at 2 weeks after injection. (**a**) Comparison of expression patterns of hASA between brains of mice given scAAV1-hASA (1.1 × 10^10^ vg) and scAAV9-hASA (1.1 × 10^10^ vg) injection. Human ASA (red) was expressed in both neurons (*labeled with NeuN, green) and astrocytes (**labeled with glial fibrillary acidic protein (GFAP), green) with scAAV1-hASA or scAAV9-hASA injection. (**b**) Comparison of hASA levels in CSF and plasma between mice given scAAV1-hASA (*n* = 3) and scAAV9-hASA (*n* = 3) injection. Error bars indicate SEM values.

To confirm that this lower transduction efficiency of scAAV9-hASA in ventricular cells was not due to technical problems with the ICV injection, we next compared the transduction efficacy between scAAV1-hASA and scAAV9-hASA when the vectors were injected into the brain parenchyma. Eight- to 9-week-old wild-type mice were given an intraparenchymal injection of 1.1 × 10^10^ vg of scAAV1-hASA (*n* = 3) or scAAV9-hASA (*n* = 3) into the right striatum. At 2 weeks after injection, immunohistochemical and biochemical analyses were performed (Fig. 3). When AAV vectors were injected into the brain parenchyma, hASA was strongly expressed in both neurons and astrocytes with either scAAV1-hASA or scAAV9-hASA injection (Fig. 3a). In addition, the CSF levels of hASA were nearly equal in mice injected with scAAV1-hASA and those injected with scAAV9-hASA (77.2 ± 24.3 vs. 65.7 ± 10.9 ng/ml, respectively) at 2 weeks after injection (Fig. 3b).

The above results suggest that the ability of scAAV1 to provide a source of therapeutic protein within the CNS was nearly equal to that of scAAV9 if they were injected by the intraparenchymal route (Fig. 3), but the ability of scAAV1 was much higher than that of scAAV9 if they were injected by the ICV route (Fig. 2). Because previous studies reported that gene therapy using AAV vector injection into the brain parenchyma reduces sulfatide levels in the brains of MLD mice^13^,^14^,^15^,^16^, we expected that ICV injection, which is considered safer than intraparenchymal injection, of scAAV1-hASA would also reduce sulfatide levels in MLD mice.

### ICV injection of scAAV1 encoding hASA induces humoral immunity and results in a slight reduction in sulfatide levels in the brains of MLD mice

**Figure 4:**
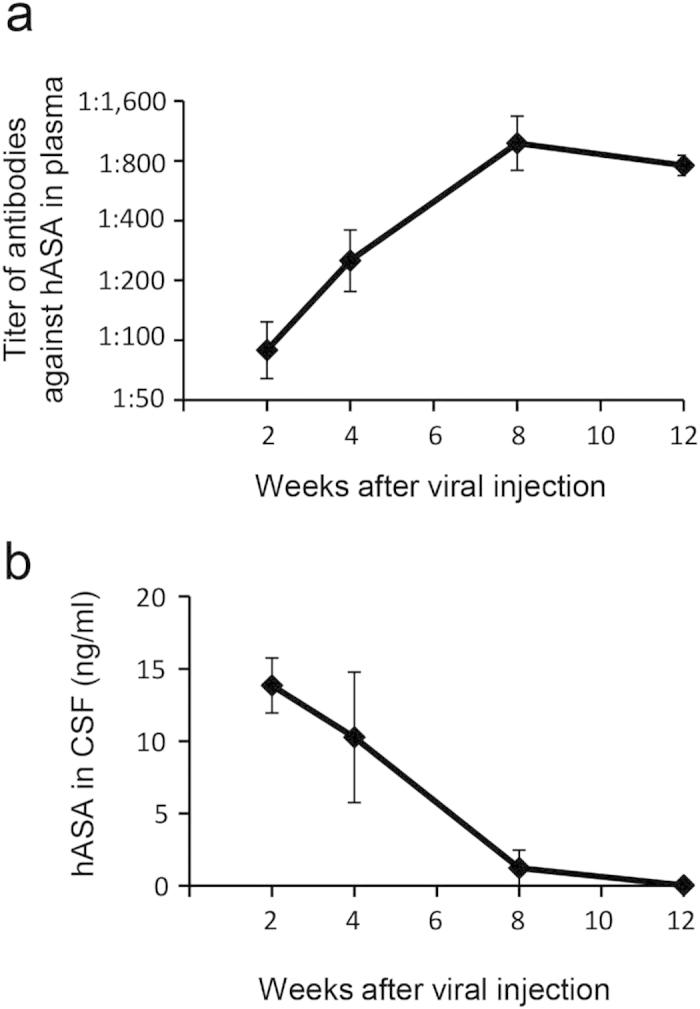
Humoral immunity developed in blood and inhibited long-term secretion of hASA into the CSF of treated MLD mice. Development of anti-hASA antibody titers in plasma (**a**) and consequent inhibition of hASA secretion into the CSF (**b**) of treated MLD mice. Blood and CSF samples were taken from mice at 2 weeks (*n* = 3), 4 weeks (*n* = 3), 8 weeks (*n* = 3), and 12 weeks (*n *= 12) after scAAV1-hASA injection. Error bars indicate SEM values.

Our previous study revealed that sulfatide is detectable in MLD mice from the age of 16 weeks^17^, and thus, we decided to start treating the mice after sulfatide accumulation had begun. Thus, 18-week-old MLD mice were given a unilateral ICV injection of 2.3 × 10^11^ vg of scAAV1-hASA (*n* = 21) as a therapeutic vector or scAAV1 encoding EGFP (scAAV1-EGFP; *n* = 5) as a control vector. Blood plasma and CSF samples were collected from different scAAV1-hASA-injected mice at 2 weeks (*n* = 3), 4 weeks (*n* = 3), 8 weeks (*n* = 3), and 12 weeks (*n* = 12) after injection and analyzed biochemically to monitor the relationship between the titer of anti-hASA antibodies in plasma and the concentration of hASA in the CSF (Fig. 4). Prior to vector injection, the mice were given intraperitoneal injection of purified hASA twice in the neonatal period for tolerization to hASA, which should allow long-term replacement of hASA in their CSF^10^. Contrary to what we expected, however, the development of anti-hASA antibodies was observed in the plasma of all treated MLD mice even at 2 weeks after ICV injection of scAAV1-hASA (Fig. 4a). Consequently, the concentrations of hASA in the CSF gradually decreased from 2 to 8 weeks and were undetectable at 12 weeks after viral injection (Fig. 4b).

**Figure 5:**
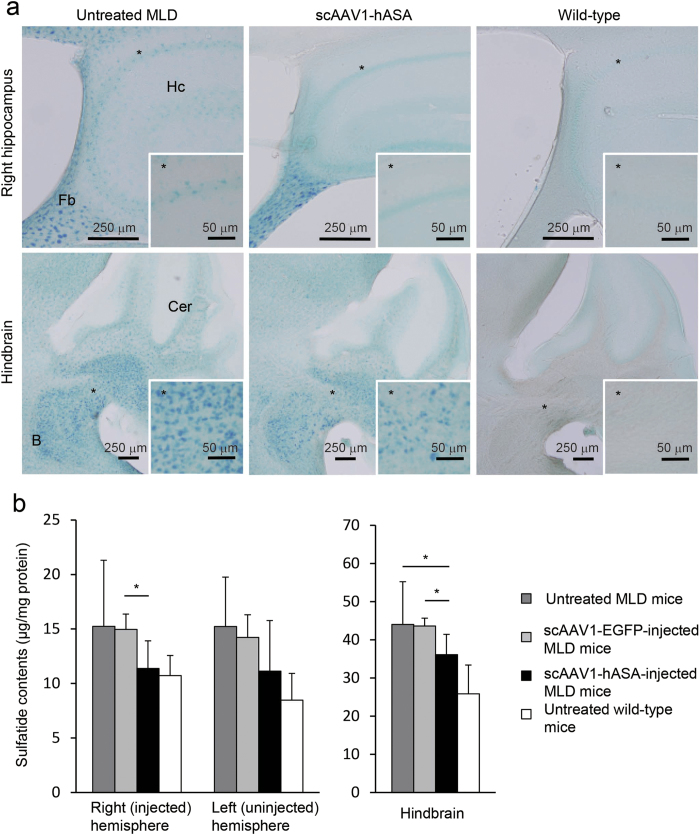
An ICV injection of scAAV1-hASA slightly reduced sulfatide levels in the brains of MLD mice. (**a**) Comparison of sulfatide deposits identified with Alcian blue staining in brains of a 30-week-old MLD mouse given scAAV1-hASA (2.3 × 10^11^ vg) injection at 18 weeks old, age-matched untreated MLD mouse, and age-matched untreated wild-type mouse. Insets are higher magnification images of the indicated (*) areas. B, brain stem; Cer, cerebellum; Fb, fimbria; Hc, hippocampus. (**b**) Comparison of sulfatide contents determined with thin-layer chromatography and normalized to protein contents in the indicated regions of brain in 30-week-old MLD mice given scAAV1-hASA (2.3 × 10^11^ vg) injection at 18 weeks old (*n* = 9), age-matched MLD mice given scAAV1-EGFP (2.3 × 10^11^ vg) injection at 18 weeks old (*n* = 3), age-matched untreated MLD mice (*n* = 12), and age-matched untreated wild-type mice (*n* = 8). Error bars indicate SD values. The asterisks indicate significant differences between scAAV1-hASA-injected and scAAV1-EGFP-injected or untreated MLD mice (*P < 0.05, Student’s t-test).

As a result, at 12 weeks after viral injection, we could detect only a slight therapeutic effect by measuring the sulfatide levels in the brain tissues of treated MLD mice compared with scAAV1-EGFP-injected MLD mice, age-matched (30 weeks old) untreated MLD mice, or age-matched untreated wild-type mice (Fig. 5). Figure 5a shows Alcian blue staining in the brain sections of treated and untreated 30-week-old mice. Compared with age-matched untreated MLD mice, sulfatide accumulation in the brain of scAAV1-hASA-injected MLD mice was slightly reduced in the hippocampus and cerebellar peduncle. Figure 5b shows sulfatide contents, which were normalized to protein contents, in the brain of scAAV1-hASA-injected MLD mice (*n* = 9), scAAV1-EGFP-injected MLD mice (*n* = 3), untreated MLD mice (*n* = 12), and untreated wild-type mice (*n* = 8). In the mice injected with scAAV1-hASA, the sulfatide contents in three brain segments (right (injected) hemisphere, left (uninjected) hemisphere, and hindbrain) were somewhat reduced compared with those of untreated MLD mice. Significant differences in sulfatide contents were observed between scAAV1-hASA-injected and scAAV1-EGFP-injected MLD mice in the right (injected) hemisphere (11.4 ± 2.5 vs. 15.0 ± 1.4 μg/mg protein, respectively; *P* < 0.05, Student’s *t*-test) and in the hindbrain (36.1 ± 5.3 vs. 43.6 ± 2.0 μg/mg protein, respectively; *P* < 0.05) and between scAAV1-hASA-injected and untreated MLD mice in the hindbrain (36.1 ± 5.3 vs. 44.0 ± 11.2 μg/mg protein, respectively; *P* < 0.05).

In this experiment, immunotolerance could not be efficiently induced, probably due to the rapid development of antibodies against the strongly induced hASA in the brain via ICV injection of scAAV1-hASA, and therefore, secretion of hASA decreased in the CSF of immune competent MLD mice, and detection of hASA was only observed for a short period. Consequently, accumulation of sulfatide was only slightly inhibited in treated MLD mice. In a model experiment, however, we injected scAAV1-hASA into the ventricle of severe combined immunodeficiency (SCID) mice, and as expected, we observed that high levels of hASA were continuously secreted into the CSF for at least 12 weeks after injection (data not shown). Thus, if we can control the immune reaction, long-term enzyme replacement therapy can be achieved in the brain.

## Discussion

For gene therapy using AAV, choosing the most suitable serotype (and type of genome packaging) in relation to the administration route and the target cells is crucial. Here, we demonstrated that the hASA level in the CSF after ICV injection of scAAV1-hASA was much higher than the levels with ssAAV1-hASA or scAAV9-hASA. However, the sulfatide levels in brain tissues injected with scAAV1-hASA were only slightly reduced compared with levels in untreated MLD mice, probably because of development of anti-hASA antibodies that inhibited the secretion of hASA into the CSF. These results suggest that this approach is potentially promising for treating MLD, but that controlling the immune response is crucial for long-term expression of hASA in the CSF.

In our experiments, we adopted the ICV route rather than the intraparenchymal route to administer AAV vectors into the CSF to transduce the ependymal cells and choroid plexus. We believe that ependymal cells and/or the choroid plexus are the preferred target for AAV-mediated gene therapy for the following two reasons. First, they are susceptible to some AAV vectors^7^,^18^,^19^ and may potentially serve as a reservoir for therapeutic proteins that are continuously released into the CSF circulating throughout the brain^7^,^10^. Raising the levels of ASA in the CSF of MLD patients may be critical for treating this disease because reconstitution of ASA activity in the CSF was shown in a patient who received hematopoietic stem cell gene therapy^20^; progression of neurodegeneration was arrested in this patient. Second, gene transfer to these tissues may be safer than to neurons or astrocytes, because we do not know how higher brain functions may be affected in human patients when the neurons and astrocytes are virally transduced and express nonphysiological, high levels of lysosomal enzyme.

In the first part of this study, we compared the transduction efficacy between ICV injection of scAAV1 and ssAAV1 vectors encoding hASA. The expression of hASA was stronger in brains injected with scAAV1-hASA than in brains injected with ssAAV1-hASA at 2 weeks after injection (Fig. 1). Together with our recent report demonstrating that even ssAAV1-hASA produces strong, stable expression of hASA in ependymal cells beyond 6 weeks after ICV injection^10^, our results suggest that ICV-injected scAAV1 vector can produce several times faster transgene expression in ependymal cells than the ssAAV1 vector. This is expected because previous studies reported that the ssAAV vector requires host cell synthesis of the complementary strand for transduction, whereas the scAAV vector does not require this step and expresses the transgene more rapidly^12^,^21^,^22^,^23^.

In the second part of this study, we compared the transduction efficacy between ICV injection of scAAV1 and scAAV9 encoding hASA. The characterization of multiple serotypes of AAV has further strengthened its use for gene therapy^18^,^19^,^24^,^25^,^26^,^27^. At 2 weeks after injection, the expression of hASA was stronger in brains injected with scAAV1-hASA compared to brains injected with scAAV9-hASA, but the plasma levels of hASA were somewhat higher in mice injected with scAAV9-hASA than in mice given scAAV1-hASA (Fig. 2). On the other hand, the amounts of hASA expression in the brain parenchyma and the CSF between mice given intrastriatal injection of scAAV1-hASA and scAAV9-hASA were nearly equal (Fig. 3). These results suggest that AAV9 vector delivered into the CSF may move more easily from the CSF space to systemic circulation than the AAV1 vector, due to the well-known blood-brain barrier permeability of AAV9^6^,^28^. As previously reported, gene therapy using intra-CSF administration of AAV9 may be effective for whole-body correction of LSDs^6^, but intra-CSF administration of AAV1 is limited to the correction of CNS pathology and is expected to be more effective for CNS pathology due to its impermeability to tissues outside the CSF compared to AAV9.

Controlling the immune response is also crucial for LSD patients receiving ERT, because therapeutic enzymes are potentially immunogenic^29^,^30^. For example, Pompe disease patients who completely lack endogenous acid alpha-glucosidase typically mount a strong immune response to injected acid alpha-glucosidase, but can be successfully treated with ERT in combination with rituximub, methotrexate, and gammaglobulins^31^,^32^. In this study, we did not observe long-term secretion of hASA into the CSF of scAAV1-injected MLD mice, probably due to the rapid development of antibodies against strongly induced hASA expression (Fig. 4). This is in contrast to our previous study using ssAAV1-hASA^10^. Consequently, compared to age-matched untreated MLD mice, we observed only a slight reduction in sulfatide content in the brain tissues of MLD mice injected with scAAV1-hASA (Fig. 5). Therefore, we believe that this treatment will be more effective if concentrations of hASA in the CSF are maintained for long period. Thus, our next step is to try a combination of AAV-mediated gene therapy with immunosuppressive drugs using animal models of LSD. However, hASA secreted into the CSF in MLD patients may not induce an immune response because most patients express mutant hASA that lacks normal functionality, but contains polypeptides that could potentially confer immunological tolerance to substituted normal hASA^33^. In fact, a recent report noted that MLD patients who received hematopoietic stem cell gene therapy are unlikely to mount an immune response toward expressed hASA^20^. In addition, a phase I/II clinical trial of intrathecal ERT for MLD patients is currently ongoing (NCT01510028; http://clinicaltrials.gov), and we will be interested to see the result of the immune response in these patients.

## Methods

### AAV vector preparation

The recombinant single-stranded AAV vector plasmid pAAV.CAhASABE, which contains the C-terminal Flag-tagged human ASA cDNA driven by the CAG promoter, was prepared as described^10^,^15^,^17^,^34^. The recombinant self-complementary (sc or double-stranded: ds) AAV vector plasmid, pdsAAV-CB-EGFP, was a kind gift from Dr. A. Srivastava (University of Florida College of Medicine, Gainesville, FL, USA). The self-complementary pdsAAV-CAGS-EGFP plasmid was generated by replacing the CBA promoter region of pdsAAV-CB-EGFP with the CAGS promoter region of pAAV.CAhASABE using the In-Fusion HD Cloning Kit (Clontech Laboratories, Inc. TaKaRa Bio, Siga, Japan). After the SalI fragment (EGFP) of pdsAAV-CAGS-EGFP was also replaced with the SalI-XhoI fragment (hASA gene) of pAAV.CAhASABE, we obtained the resulting self-complementary plasmid, pdsAAV-CAGS-hASA. To generate the AAV1 or AAV9 vector, an adenovirus-free triple transfection method was used as described^10^,^15^,^17^,^34^,^35^ with packaging plasmids Rep2/Cap1 or Rep2/Cap9. The AAV vg titer was determined as described^10^.

### Animals and injections

All animal experiments were approved by the Ethics Committee of Nippon Medical School and were carried out according to the institutional guidelines for animal care (Nippon Medical School, Tokyo, Japan). ASA knockout (MLD) mice, which are on the C57BL/6J genetic background following backcrossing of F1 hybrid mice^36^, were obtained from the laboratory of Dr. V. Gieselmann (University of Bonn, Bonn, Germany). Wild-type (C57BL/6J) and SCID mice were purchased from Saitama Experimental Animals Supply Co. (Saitama, Japan). To reduce the effect of antibody development against hASA in the AAV-injected animals, we attempted to immunotolerize MLD mice to hASA using a published protocol in which neonatal mice were intraperitoneally injected with 10 μg of enzyme protein 30–40 h and 5 days after birth^10^,^37^. To assess the effectiveness of treatment, we used age-matched untreated MLD mice (*n* = 13), including tolerized MLD mice (intraperitoneally injected with hASA during the neonatal period; *n* = 4) and MLD mice that were not tolerized (no injection of hASA; *n* = 9). The sulfatide levels in brain tissues were not significantly different between these two groups (data not shown).

To administer the vector, the mice were anesthetized by intraperitoneal injection of pentobarbiturate (50 mg/kg) and positioned on a stereotactic frame (SR-6N; Narishige, Tokyo, Japan). The skin over the skull was incised, and a small hole was made in the skull above the target using a microdrill. The stereotactic coordinates were as follows: anteroposterior (AP), −0.4 mm; mediolateral (ML), −1.0 mm; dorsoventral (DV), −3.0 mm from the bregma for injection into the right lateral ventricle; and AP, +1.0 mm; ML, −2.0 mm; DV, −4.0 mm from the bregma for injection into the right striatum. Animals were injected unilaterally with 20 μl (for injection into the right lateral ventricle) or 2 μl (for injection into the right striatum) using a syringe with a 0.52 mm needle (Ito Co., Shizuoka, Japan). Vector was injected over 10 min, and the needle was left in place for 10 min prior to withdrawal.

### Collection of CSF and blood

At various times after injection, mice were deeply anesthetized and transcardially perfused with 0.1 M phosphate-buffered saline (PBS). Prior to perfusion, CSF samples were taken from the cisterna magna using a previously reported puncture technique^38^, and blood was collected by cardiac puncture. The volume of CSF and blood obtained from each mouse was 4–10 μl and 400–500 μl, respectively. CSF samples with visible blood contamination were discarded. After whole blood was treated with heparin and centrifuged, the resulting supernatant was recovered as a plasma sample.

### Histology

After perfusion fixation, the brains were dissected and postfixed overnight in 4% paraformaldehyde at 4 °C before immersion in PBS containing 30% sucrose for cryoprotection. Brain sections were then cut to a thickness of 20 μm for immunostaining or 100 μm for Alcian blue staining using a freezing sliding microtome. For immunohistochemical analyses, free-floating sections (20 μm thick) were washed in PBS and then blocked for 1 h in 1% skim milk (Nacalai Tesque, Kyoto, Japan) in PBS. Immunostaining was performed as described^10^ and using the following antibodies: anti-hASA goat IgG (1:100; R&D Systems, Minneapolis, MN, USA), anti-NeuN mouse IgG (1:500; Chemicon International, Temecula, CA, USA) and anti-glial fibrillary acidic protein mouse IgG (1:500; Sigma-Aldrich, St Louis, MO, USA) as primary antibodies, and biotinylated anti-goat rabbit IgG (1:100; Vector Laboratories, Burlingame, CA, USA) and Cy3-conjugated affinipure anti-mouse donkey IgM (1:100; Jackson ImmunoResearch, West Grove, PA, USA) as secondary antibodies. Fluorescent signals were imaged using a BX-60 fluorescence microscope (Olympus, Tokyo, Japan). To detect sulfatides, brain sections (100 μm thick) were stained with Alcian blue (Sigma-Aldrich) as described^39^.

### Biochemical analysis

Concentrations of hASA in CSF and plasma were determined using an indirect sandwich ELISA as described^10^,^17^. For the ELISA, 2 μl of CSF or plasma at a 1:30 dilution gave a satisfactory result. No hASA was detected in CSF or plasma from untreated (AAV vector was not injected) mice (data not shown).

The titer of anti-hASA antibody in plasma was determined by measuring the capacity of a 2-fold dilution series of 2-μl aliquots of plasma to immunoprecipitate hASA as described^10^. The value of the titer described in this report represents the calculated plasma dilution at a turning point in the sigmoid function, which was fitted to the dilution curve obtained experimentally as described^10^.

Sulfatide levels were determined as follows. The excised brains were separated into three segments (right and left hemispheres and hindbrain) and homogenized in pure water. Homogenates were centrifuged at 15,000 rpm for 10 min at 4 °C. The supernatants were collected for determination of the protein content with the Bio-Rad DC Assay (Bio-Rad, Hercules, CA, USA) and sulfatide content with thin-layer chromatography. Crude lipids were extracted from tissue homogenates using the Folch method^40^. Briefly, lipids were successively extracted with mixtures of chloroform/methanol (1:2, vol:vol), chloroform/methanol (1:1, vol:vol), and chloroform/methanol/water (86:14:1, vol:vol:vol). Glycosphingolipids recovered in Folch’s lower phase were dried under a stream of nitrogen and treated with mild alkaline solution (0.1 M NaOH in methanol) at 40 °C for 1  h. After neutralizing the solution with 1 M acetic acid, glycosphingolipids were again recovered in Folch’s lower phase and applied to precoated high-performance thin-layer chromatography silica gel 60F254 plates (100 × 200 mm, Merck KGaA, Darmstadt, Germany). Plates were developed in chloroform/methanol/water (70:30:4, vol:vol:vol). After development, plates were spread with 10% (w/v) cupric sulfate hydrate in 8% (v/v) phosphoric acid solution, and then charred at 180°C for 10 min. Amounts were quantitatively determined with densitometric scanning using CS Analyzer 3 (ATTO, Tokyo, Japan). Standard sulfatide was obtained from Wako Pure Chemical Industries (Osaka, Japan). The obtained sulfatide content was normalized to the protein content and represented as μg/mg protein.

### Statistical analysis

Data are expressed as the mean ± standard error of the mean (SEM) or standard deviation (SD) as described in the individual figure legends. Each data set was treated statistically as described in the individual figure legends. An error level of 5% (*P* < 0.05) was considered significant.

## Additional Information

**How to cite this article**: Hironaka, K. *et al.* Enzyme replacement in the CSF to treat metachromatic leukodystrophy in mouse model using single intracerebroventricular injection of self-complementary AAV1 vector. *Sci. Rep.* **5**, 13104; doi: 10.1038/srep13104 (2015).
